# Feasibility, endocrine and anti-tumour effects of a triple endocrine therapy with tamoxifen, a somatostatin analogue and an antiprolactin in post-menopausal metastatic breast cancer: a randomized study with long-term follow-up.

**DOI:** 10.1038/bjc.1998.18

**Published:** 1998

**Authors:** M. Bontenbal, J. A. Foekens, S. W. Lamberts, F. H. de Jong, W. L. van Putten, H. J. Braun, J. T. Burghouts, G. H. van der Linden, J. G. Klijn

**Affiliations:** Division of Endocrine Oncology (Department of Medical Oncology), Dr Daniel den Hoed Kliniek, Rotterdam, The Netherlands.

## Abstract

Suppression of the secretion of prolactin, growth hormone and insulin-like growth factor 1 (IGF-1) might be important in the growth regulation and treatment of breast cancer. Because oestrogens may counteract the anti-tumour effects of such treatment, the combination of an anti-oestrogen (tamoxifen), a somatostatin analogue (octreotide) and a potent anti-prolactin (CV 205-502) might be attractive. In this respect, we performed a first exploratory long-term study on the feasibility of combined treatment and possible clear differences in endocrine and anti-tumour effects during such combined treatment vs standard treatment with tamoxifen alone. Twenty-two post-menopausal patients with metastatic breast cancer (ER and/or PR positive or unknown) were randomized to receive either 40 mg of tamoxifen per day or the combination of 40 mg of tamoxifen plus 75 microg of CV 205-502 orally plus 3 x 0.2 mg of octreotide s.c. as first-line endocrine therapy. An objective response was found in 36% of the patients treated with tamoxifen alone and in 55% of the patients treated with combination therapy. Median time to progression was 33 weeks for patients treated with tamoxifen and 84 weeks for patients treated with combination therapy, but the numbers are too small for hard conclusions. There was no difference in overall post-relapse survival between the two treatment arms. With respect to the endocrine parameters, there was a significant decrease of plasma IGF-1 levels in both treatment arms, whereas during combined treatment plasma growth hormone tended to decrease and plasma prolactin levels were strongly suppressed; in some patients insulin and transforming growth factor alpha (TGF-alpha) decreased during the triple therapy. Although there was no significant difference in mean decrease of plasma IGF-1 levels between the two treatment arms, combined treatment resulted in a more uniform suppression of IGF-1. Therefore, the addition of a somatostatin analogue and an anti-prolactin may potentially enhance the efficacy of anti-oestrogens in the treatment of breast cancer owing to favourable endocrine and possible direct anti-tumour effects. Large phase III trials using depot formulations (to increase the feasibility) of somatostatin analogues are warranted to demonstrate the potential extra beneficial anti-tumour effects of such combination therapy.


British Journal of Cancer (1998) 77(1), 115–122
© 1998 Cancer Research Campaign

### Feasibility, endocrine and anti-tumour effects of a triple endocrine therapy with tamoxifen, a somatostatin analogue and an antiprolactin in post-menopausal metastatic breast cancer: a randomized study with long-term follow-up

**M Bontenbal[1], JA Foekens[1], SWJ Lamberts[2], FH de Jong[2], WLJ van Putten[3], HJ Braun[4], JThM Burghouts[5], GHM van der Linden[6] and JGM Klijn[1]**

[1]Division of Endocrine Oncology (Department of Medical Oncology) and [3]Department of Statistics, Dr Daniel den Hoed Kliniek; [2]Department of Internal Medicine III and Endocrinology, Erasmus University, Rotterdam; [4]Schielandhospital, Schiedam: [5]Groot Ziekengasthuis, Den Bosch: [6]Refaja Hospital, Dordrecht, The Netherlands

**Summary** Suppression of the secretion of prolactin, growth hormone and insulin-like growth factor 1 (IGF-1) might be important in the growth regulation and treatment of breast cancer. Because oestrogens may counteract the anti-tumour effects of such treatment, the combination of an anti-oestrogen (tamoxifen), a somatostatin analogue (octreotide) and a potent anti-prolactin (CV 205–502) might be attractive. In this respect, we performed a first exploratory long-term study on the feasibility of combined treatment and possible clear differences in endocrine and anti-tumour effects during such combined treatment vs standard treatment with tamoxifen alone. Twenty-two post-menopausal patients with metastatic breast cancer (ER and/or PR positive or unknown) were randomized to receive either 40 mg of tamoxifen per day or the combination of 40 mg of tamoxifen plus 75 μg of CV 205–502 orally plus 3 × 0.2 mg of octreotide s.c. as first-line endocrine therapy. An objective response was found in 36% of the patients treated with tamoxifen alone and in 55% of the patients treated with combination therapy. Median time to progression was 33 weeks for patients treated with tamoxifen and 84 weeks for patients treated with combination therapy, but the numbers are too small for hard conclusions. There was no difference in overall post-relapse survival between the two treatment arms. With respect to the endocrine parameters, there was a significant decrease of plasma IGF-1 levels in both treatment arms, whereas during combined treatment plasma growth hormone tended to decrease and plasma prolactin levels were strongly suppressed; in some patients insulin and transforming growth factor α (TGF-α) decreased during the triple therapy. Although there was no significant difference in mean decrease of plasma IGF-1 levels between the two treatment arms, combined treatment resulted in a more uniform suppression of IGF-1. Therefore, the addition of a somatostatin analogue and an anti-prolactin may potentially enhance the efficacy of anti-oestrogens in the treatment of breast cancer owing to favourable endocrine and possible direct anti-tumour effects. Large phase III trials using depot formulations (to increase the feasibility) of somatostatin analogues are warranted to demonstrate the potential extra beneficial anti-tumour effects of such combination therapy.

**Keywords:** breast cancer; GH/IGF-1 axis: somatostatin analogue; anti-prolactin

Different steroid hormones, peptide hormones, growth factors and other trophic substances are involved in the growth regulation of human breast cancer (Clarke et al, 1992; Klijn et al, 1992). Oestrogens, especially oestradiol, are the most potent growth stimulatory hormones of breast cancer. Therefore, endocrine treatment of metastatic breast cancer usually uses antisteroidal agents such as tamoxifen, resulting in response rates of 30–40% (Santen et al, 1990).

Together with oestradiol, insulin-like growth factors (IGF-1 and IGF-2) are the most potent mitogens for breast cancer cells (Osborne et al, 1990; Clarke et al, 1992; Cullen et al, 1992). The growth effects of both are mediated predominantly via IGF-1 receptors, which have been demonstrated in 67–93% of primary human breast cancers (Pekonen et al, 1988; Peyrat et al, 1988*a*; Foekens et al, 1989*a*; Klijn et al, 1993) at higher density than in normal or benign breast tissue (Peyrat et al, 1988*b*). In vivo, pituitary-derived growth hormone (GH) regulates endocrinologically the secretion of IGF-1 (Kelly et al, 1991; Lamberts et al, 1991), but possibly also has regulatory effects on local IGF-1 secretion within (tumour) tissues (Davoren et al, 1986; Schally et al, 1987; Kelly et al, 1991). In addition, in breast cancer local production of GH with a potential paracrine function has been described (Mol et al, 1995). In vitro, physiological concentrations of the lactotrophic hormones GH and prolactin (PRL) can stimulate the growth of breast cancer cells (Malarkey et al, 1983; Murphy et al, 1984; Manni et al, 1986; Bonneterre et al, 1990). In primary human breast cancers, receptors for these lactotrophic hormones have been demonstrated in 13–72% of series of tumours investigated depending on the techniques used (Bonneterre et al, 1990). Furthermore, increased plasma levels of both GH (Emerman et al,

*Received 23 January 1997*
*Revised 26 June 1997*
*Accepted 11 July 1997*

*Correspondence to:* JGM Klijn, Division of Endocrine Oncology, Rotterdam Cancer Institute/Dr Daniel den Hoed Kliniek, Groene Hilledijk 301, 3075 EA Rotterdam, The Netherlands

**Table 1** Patient characteristics

| | Tamoxifen | Combination therapy | Total |
|---|---|---|---|
| Number of patients entered | 12 | 10 | 22 |
| Number of patients evaluable | 11 | 9 | 20 |
| Menopausal status: | | | |
| Post | 10 | 9 | 19 |
| Peri | 1 | 0 | 1 |
| Age | | | |
| Mean (range) | 59 (49–71) | 62 (49–73) | 60 (49–73) |
| WHO performance status | | | |
| 0 | 6 | 5 | 11 |
| 1 | 3 | 2 | 5 |
| 2 | 2 | 2 | 4 |
| Disease sites | | | |
| Soft tissue | 3 | 3 | 6 |
| Lymph nodes | 1 | 1 | 2 |
| Bone | 9 | 6 | 15 |
| Liver | 4 | 1 | 5 |
| Lung | 2 | 3 | 5 |
| Number of disease sites | | | |
| 1 | 5 | 5 | 10 |
| 2 | 5 | 3 | 8 |
| 3 | 0 | 1 | 1 |
| 4 | 1 | 0 | 1 |
| Receptor status (in tumour or metastases): | | | |
| ER and/or PR positive | 10 | 6 | 16 |
| ER and PR unknown | 1 | 3 | 4 |

1985) and PRL (Holtkamp et al, 1984; Emerman et al, 1985) as well as of IGF-1 (Peyrat et al, 1993) have been found in patients with breast cancer. Therefore, suppression of GH, PRL and IGF-1 secretion might be important in the treatment of breast cancer.

Suppression of GH and IGF-1 secretion can be induced by somatostatin and its analogues (Schally et al, 1987; Schally et al, 1988; Manni et al, 1989, Pollak et al, 1989; Lamberts et al, 1991; Lamberts et al, 1996). Interestingly, receptors for somatostatin (SSTR) have also been demonstrated in 36–67% of primary human breast cancers (Reubi et al, 1990; Fekete et al, 1989; Klijn et al, 1992; 1993) and in even 75% by in vivo receptor scintigraphy (Van Eijck et al, 1994), indicating that somatostatin analogues can directly affect tumour growth. Indeed, we (Setyono-Han et al, 1987) and others have previously shown (Lamberts et al, 1991; Weckbecker et al, 1992) direct growth-inhibitory effects of somatostatin analogues on human breast cancer cell lines.

Based on the data mentioned above, it can be concluded that somatostatin analogues and antiprolactins can have beneficial direct and indirect effects on the treatment of breast cancer. However, until now a single treatment with these agents showed only minor activity in post-menopausal patients with metastatic breast cancer (European Breast Group, 1972; Minton et al, 1974; Engelsman et al, 1975; Grisoli et al, 1981; Morten et al, 1988; Manni et al, 1989; Vennin et al, 1989; Holtkamp et al, 1990; Klijn et al, 1992). Because unopposed oestrogen action can overrule the growth inhibitory effects of somatostatin analogues (Setyono-Han et al, 1987) and/or anti-prolactions, combination treatment with an anti-oestrogen, a somatostatin analogue and an anti-prolactin might be of value and may increase the efficacy of single treatment

**Table 2** Mean plasma levels of hormones and growth factors before and during treatment

| Hormone/ growth factor | Treatment | Number of evaluable patients (*n*) | Pretreatment value (mean ± s.d.) | Absolute change from pretreatment value (mean ± s.d.) |
|---|---|---|---|---|
| E2 | TAM | 7 | 87 ± 75 | – 21 ± 63 |
| (pmol $l^{-1}$) | Combined | 6 | 83 ± 80 | – 17 ± 74 |
| | | | | *P* = 0.48* |
| nGH | TAM | 10 | 0.8 ± 1.2 | + 1.23 ± 1.78 |
| (μg $l^{-1}$) | Combined | 7 | 2.6 ± 3.1 | – 1.3 ± 3.32 |
| | | | | *P* = 0.10 |
| PRL | TAM | 10 | 5.2 ± 1.9 | – 0.77 ± 2.62 |
| (μg $l^{-1}$) | Combined | 7 | 8.0 ± 5.2 | – 5.5 ± 4.85 |
| | | | | *P* = 0.006 |
| Insulin | TAM | 10 | 25.7 ± 15.3 | + 15.6 ± 39.8 |
| (mU $l^{-1}$) | Combined | 7 | 57.5 ± 41.4 | – 32 ± 37.6 |
| | | | | *P* = 0.02 |
| TGF-α | TAM | 10 | 0.30 ± 0.14 | + 0.02 ± 0.09 |
| (ng $ml^{-1}$) | Combined | 8 | 0.39 ± 0.13 | – 0.08 ± 0.12 |
| | | | | *P* = 0.11 |
| IGF-1 | TAM | 10 | 149 ± 64 | – 62 ± 47 |
| (ng $ml^{-1}$) | Combined | 8 | 137 ± 39 | – 69 ± 28 |
| | | | | *P* = 0.63 |

**P*-values indicate differences in decrease between the two treatment groups. TAM, tamoxifen; Combined, combination treatment with tamoxifen, octreotide and CV 205–502.

© Cancer Research Campaign 1998

**Figure 1** Effect of tamoxifen (left) and of combination treatment (right) on plasma hormone and growth factor concentrations. The zero lines represent the basal pretreatment values, whereas the absolute individual changes are indicated as determined 4–24 weeks after the start of treatment with tamoxifen alone. As tamoxifen affects growth factor secretion (Coletti et al, 1989; Pollak et al, 1990; Clarke et al, 1992; Kiang et al, 1992; Lönning et al, 1992; Reed et al, 1992; Winston et al, 1994) such combination treatment might be extra attractive. However, clinical results of such combined treatment modality have not yet been reported. In this paper, we report on the feasibility, the endocrine and long-term anti-tumour effects of combined treatment with tamoxifen, the somatostatin analogue octreotide and a new potent dopamine agonist (the anti-prolactin CV 205–502) in comparison with those of single treatment with tamoxifen, as well as an in-depth discussion about the mechanisms of action and an elaborate overview of literature data.

### PATIENTS AND METHODS

The study was performed after approval by a local Human Investigations Committee (trial DDHK 88–30). Between August 1989 and May 1991, 22 post-menopausal patients with previously untreated metastatic breast cancer were randomized to be treated within this trial after previous informed consent. The patients characteristics are summarized in Table 1. Two patients were not evaluable: one stopped treatment with octreotide within 2 days because the patient could not tolerate daily injections and another patient stopped single treatment with tamoxifen within 2 months because of the detection of an endometrial carcinoma. Later on, one patient appeared to be perimenopausal because of a rise in oestradiol levels after the start of treatment with tamoxifen. Therefore, this patient was not included in the analysis for oestradiol levels. Currently, the mean follow-up of all 20 evaluable patients is 3 years (range 3 months–6 years). Within this follow-up period all but two patients showed progressive disease and 14 died.

The patients were randomized to be treated with either tamoxifen 40 mg per day or with the combination treatment consisting of 40 mg of tamoxifen, 75 μg of the dopamine agonist CV 205–502 (Norprolac) and the somatostatin analogue octreotide (Sandostatin) 200 mg t.i.d. subcutaneously every day. Dose modification was not allowed. The duration of treatment varied from 6 weeks to more than 6 years. Patients were evaluated for toxicity and response every 6–12 weeks. Measurements of tumour response were performed according to the UICC criteria.

Plasma samples for measurement of basal hormone and growth factor concentrations (Table 2) were taken before and regularly between 4–24 weeks after start of treatment (Figure 1). Plasma peptide hormones and growth factors were measured by radioimmunoassays and radioreceptor assay (TGF-α), as described previously (Klijn et al, 1990*a*). Plasma oestradiol levels were measured by radioimmunoassay.

### Statistical methods

The expected accrual rate per year was 60 patients. Because of a much lower actual recruitment, in particular because of the refusal of daily injections in the combined treatment arm, the trial was closed after the inclusion of 22 patients in 2 years. Because of the relatively low number of patients in this study, the analysis of the data has been primarily descriptive, directed at the calculation of response rate, progression-free survival with actuarial methods and a description of the endocrine effects of the treatments by calculating the change in plasma concentration levels from the baseline. Because of the limited power of this study to detect differences between treatment arms, all *P*-values reported in this paper should be regarded as exploratory. The log-rank test was used for the comparison of progression free survival. The Mann–Whitney non-parametric two-sample test was used to compare the change in plasma levels in both treatment groups.

**Table 3** Type of responses and time to progression (in weeks)

| | CR | PR | SD | PD |
|---|---|---|---|---|
| Tam | 1 | 3 | 3 | 4 |
| | (162) | (32,66,78) | (25,39,159) | (10–21) |
| Combination | 2 | 3 | 2 | 2 |
| treatment | (171,209) | (84,86,115) | (22,36) | (7,11) |

### RESULTS

### Endocrine effects of treatment

Figure 1 shows the absolute change from baseline in plasma hormone and growth factor concentrations for all patients with evaluable measurements. As no trend was apparent in the values during treatment from 1 month after the start of treatment, for each patient all these values measured between 4 and 24 weeks are summarized by the mean. Table 2 shows the mean pretreatment values and the absolute mean change of each of the endocrine parameters from pretreatment values. Pretreatment basal oestradiol levels were similar in both treatment groups and the values during treatment did not show a systematic change. Basal GH showed a small decrease in four out of seven investigated patients during combined treatment and in none of the ten patients during single treatment with tamoxifen (Figure 1), but in view of differences in pretreatment values and a large variation during treatment there was only a trend for a difference ($P = 0.10$) between the two treatment arms (Table 2). Most interesting was the significant decrease ($P < 0.0002$) of plasma IGF-1 levels during treatment (Figure 1), i.e. overall a mean decrease of 49% during combined treatment and 38% during single treatment with tamoxifen. This decrease showed no significant difference between the two treatment groups, either absolutely ($P = 0.63$, Table 2) and percentually ($P = 0.21$). However, IGF-1 suppression was more uniform during combined treatment in contrast to a strong variation in response during tamoxifen treatment (Figure 1). In plasma prolactin levels, the combined treatment caused a clearly significant suppression of prolactin secretion owing to the antiprolactin CV 205–502, whereas tamoxifen had no significant effect (Figure 1, Table 2, $P = 0.006$).

In the other endocrine parameters, some patients showed a decrease of plasma insulin and TGF-α levels during combined treatment (Figure 1), but differences in overall results between the two treatment arms (Table 2) were only found for insulin ($P = 0.02$) and not for TGF-α ($P = 0.11$).

### Anti-tumour effects

Five (55%) out of nine patients treated with the combination therapy showed an objective response compared with 4 (36%) out of 11 patients treated with tamoxifen alone (Table 3). The median time to progression was 84 weeks for the patients treated with combination therapy vs 32 weeks for patients treated with tamoxifen. Progression-free survival was slightly better for patients treated with the combination of drugs than those treated with

© Cancer Research Campaign 1998

**Figure 2** Actuarial progression-free survival curves for the two treatment groups tamoxifen alone (Figure 2), but the number of patients in this feasibility study are too few to draw definite conclusions. There was no difference between the two treatment arms with respect to overall post-relapse survival.

### Toxicity

Treatment with the triple endocrine combination therapy appeared to be feasible, but a significant number (about 40–50%) of potentially eligible patients refused randomization because of the inclusion of three daily subcutaneous injections with Sandostatin within one of the treatment arms. However, subjective side-effects were minimal in both treatment arms. During combination therapy, shortly after the start of treatment, slight nausea grade 1 (WHO) was observed in a minority of the patients, but no serious complaints were reported. One patient with diabetes mellitus had a persistent fall in plasma glucose levels during combined therapy needing the reduction of daily insulin dosages (maybe as a consequence of suppression of glucagon secretion by octreotide). The most important side-effect was the development of asymptomatic gallbladder stones in one patient treated with combination therapy.

### DISCUSSION

The relative role of PRL, GH and IGF-I in the development and treatment of human breast cancer is not clearly understood. All three peptides have been observed to be increased in plasma from a variable percentage of breast cancer patients (Holtkamp et al, 1984; Emerman et al, 1985; Peyrat et al, 1993). Growth stimulation of breast cancer cells by these peptides can be blocked by monoclonal antibodies (Pollak et al, 1988; Arteaga et al, 1989; Ginsburg et al, 1995; Mershon et al, 1995). In addition, all three peptides (Foekens et al, 1989*b*; Clarke et al, 1992; Fields et al, 1993; Ginsburg et al, 1995; Mershon et al, 1995; Mol et al, 1995) and their receptors (Pekonen et al, 1988; Peyrat et al, 1988*a*; Foekens et al, 1989*a*; Bonneterre et al, 1990; Clarke et al, 1992; Klijn et al, 1993), have been demonstrated in animal mammary tumours and/or human primary breast cancers suggesting a role in autocrine/paracrine cell growth regulation. However, nearly all endocrine therapies are focused on antagonism of oestradiol, the primary mitogen for human breast cancer (Santen et al, 1990).

Some trials have tested the value of suppression of prolactin secretion by dopamine agonists (antiprolactins) (European Breast Cancer Group et al, 1972; Minton et al, 1974; Engelsman et al, 1975; Grisoli et al, 1981; Fentimen et al, 1988; Morten et al, 1988; Manni et al, 1989; Holtkamp et al, 1990). Initial trials using single dopaminergic treatment with L-dopa or bromocriptine showed poor results (European Breast Cancer Group et al, 1972; Minton et al, 1974; Engelsman et al, 1975; Grisoli et al, 1981). Two studies investigated combination therapy of bromocriptine with antisteroidal treatment. Dogliotti et al (1987) found that bromocriptine in combination with high dose progestins reduced the percentage of patients with progressive disease, but Bonneterre et al (1988) observed no additional anti-tumour effect of bromocriptine to tamoxifen. This might be explained by the facts that progestins can increase plasma PRL levels (Alexieva-Figusch et al, 1984), whereas tamoxifen has rather inhibitory effects on PRL secretion (Klijn et al, 1985; Lamberts et al, 1990; Malaab et al, 1992). Other authors (Manni et al, 1989; Bonneterre et al, 1990; Pollak et al, 1992) assumed that the lack of anti-tumour effects by single dopaminergic treatment may have been due to the presence of hGH, which is also a lactogen and can bind to lactotrophic receptors (Bonneterre et al, 1990). However, a few pilot studies using combined treatment with bromocriptine and a GH-lowering drug, such as a somatostatin analogue, showed no impressive effects in heavily pretreated patients with metastatic breast cancer (Morten et al, 1988; Manni et al, 1989; Holtkamp et al, 1990; Klijn et al, 1992).

In view of the accumulating evidence regarding the importance of IGFs in the growth regulation of breast cancer (Osborne et al, 1990; Clarke et al, 1992), in the past decade there has been an increasing interest in the GH/IGF axis, in particular because of the development of potent somatostatin analogues, agents which can suppress the function of the GH/IGF axis (Schally et al, 1987; Schally et al, 1988; Manni et al, 1989; Klijn et al, 1990*a*; Lamberts et al, 1991, 1996). This interest was further increased by the detection of SSTRs in breast cancer cell lines and tissues (Setyono-Han et al, 1987; Srkalovic et al, 1990; Weckbecker et al, 1992; Prévost et al, 1994; Buscail et al, 1995) and in about half of primary breast cancers (Fekete et al, 1989; Reubi et al, 1990; Van Eijck et al, 1994). Indeed, we (Setyono-Han et al, 1987) and others (Lamberts et al, 1991; Weckbecker et al, 1992) have demonstrated direct growth-inhibitory effects by various somatostatin analogues on different breast cancer cell lines. Inhibition of cell proliferation seems to be mediated especially by subtypes SSTR2 and SSTR5 (Buscail et al, 1995; Lamberts et al, 1996). In addition, in some experimental animal models somatostatin analogues were able to cause inhibition of mammary tumour growth (Rose et al, 1983; Schally et al, 1987, 1988; Szende et al, 1989; Weber et al, 1989; Lamberts et al, 1991; Weckbecker et al, 1994; Lamberts et al, 1996). However, in four clinical studies (Morten et al, 1988; Manni et al, 1989; Vennin et al, 1989; Holtkamp et al, 1990), treatment of 38 (heavily) pretreated patients with octreotide caused only one objective response and five times stable disease (together 16%) (Klijn et al, 1992). In addition, in previously untreated patients single first-line treatment with octreotide appeared to be less effective than common standard treatment modalities, which resulted in early stopping of this treatment arm in an on-going randomized trial of the Mayo Clinics.

© Cancer Research Campaign 1998

These disappointing results of single somatostatin analogue treatment (or in combination with an anti-prolactin) can be explained by our observation that oestradiol abolished these growth inhibitory effects (Setyono-Han et al, 1987). Therefore, at the start of the present clinical study in 1989 our study design testing these drugs in combination with an anti-oestrogen seems to be more appropriate. Later on, this approach was supported by the results of different preclinical studies that showed the additive biological (Huynh et al, 1994) and anti-tumour effects (Weckbecker et al, 1994; Bogden et al, 1995) of somatostatin analogues to endocrine therapy with tamoxifen or by surgical oophorectomy in hormone sensitive tumours in vivo. Meanwhile, tamoxifen appeared not only to act by blocking the growth stimulatory effects of oestrogens but also to modify growth factor secretion (Coletti et al, 1989; Pollak et al, 1990; Butta et al, 1992; Clarke et al, 1992; Kiang et al, 1992; Lönning et al, 1992; Pollak et al, 1992; Reed et al, 1992; Huynh et al, 1994; Winston et al, 1994; Kopp et al, 1995; Van Roozendaal et al, 1995) and to suppress the GH/IGF-I axis (Malaab et al, 1992; Pollak et al, 1992; Tannenbaum et al, 1992) and prolactin secretion (Klijn et al, 1985; Lamberts, 1990, Malaab et al, 1992). Tamoxifen and other anti-oestrogens can decrease plasma IGF-1-levels (Coletti et al, 1989; Pollak et al, 1990; 1992; Kiang et al, 1992; Lönning et al, 1992; Reed et al, 1992; Winston et al, 1994), but also can down-regulate IGF-I-R (Freiss et al, 1990) and can suppress IGF-1-induced breast cancer cell proliferation (Pratt et al, 1993). Furthermore, both octreotide (Lamberts et al, 1991) and anti-oestrogens (Löning et al, 1992; Reed et al, 1992; Pratt et al, 1993; Winston et al, 1994) affect IGF-binding proteins. Thus, additive endocrine and anti-tumour effects could be expected from combination therapy with tamoxifen plus a somatostatin analogue and an anti-prolactin.

In our study, tamoxifen caused an increase rather than a decrease in basal GH concentrations. In contrast, in several patients combination treatment tended to decrease basal GH levels. Previously, Pollak et al (1989) and Manni et al (1989) showed significant suppression of stimulated GH-levels (which are less affected by fluctuation than basal plasma GH levels during the day) by octreotide treatment. In contrast to the basal GH concentration, IGF-I levels are stable during the day. Strikingly, we found no additive suppressive effects of tamoxifen and octreotide on mean plasma IGF-1 concentrations, but combination treatment caused a more uniform suppression of IGF-1 (Figure 1). Both single tamoxifen and combination treatment caused a decrease of about 40–50%. This might partly be explained by the observation that tamoxifen already increases the release of endogenous hypothalamic somatostatin, resulting in blunting of pituitary GH pulse amplitude (Tannenbaum et al, 1992). However, this does not exclude that clear additive endocrine effects might be found in studies using lower dosages of tamoxifen (20 instead of 40 mg day$^{-1}$) or higher dosages of somatostatin analogues than used in our trial. Recently, Kiang et al (1992) reported an interesting observation indicating that the type of anti-tumour response is related to the extent of IGF-I suppression. In our study, we were not able to confirm this observation.

In basal plasma insulin and TGF-$\alpha$ concentrations, we found no impressive differences between the two treatment arms, although the combination therapy had a suppressive effects in some patients. Although somatostatin analogues might affect LH secretion in premenopausal patients (Chiodera et al, 1986), in our post-menopausal patients no significant effects on plasma $E_2$ levels were observed. This finding confirms the results of the study of Manni et al (1989) who also found no effect of combined octreotide/bromocriptine treatment on plasma LH, FSH and $E_2$ levels. Finally, with respect to prolactin secretion (which is partly influenced by oestrogens) the anti-oestrogen tamoxifen tended to decrease plasma PRL levels, although not significantly. Interestingly, the new very potent antidopaminergic drug CV 205–502 used in the treatment of prolactinomas (Rasmussen et al, 1987) caused a strong significant decrease of basal prolactin levels (with about 70%) in our patients with normal PRL secretion. This suppression is more pronounced than previously reported for bromocriptine. However, in contrast to oestradiol, it is currently unknown to which plasma levels PRL has to be suppressed to contribute to an (potential) extra anti-tumour effect.

With respect to the performance of our study, the triple endocrine therapy appeared to be feasible in the presence of only few non-serious side-effects. However, a significant number (40–50%) of potentially eligible patients refused participation in the trial because of the need of three daily injections in one of the treatment arms. This problem will be resolved by the application of depot preparations of somatostatin analogues that are increasingly available. Furthermore continuous administration of drugs is generally more effective than daily injections as demonstrated for octreotide (Klijn et al, 1990*b*; Weckbecker et al, 1994). In our pilot study, the patients treated with the combination therapy showed progressive disease from start of treatment less frequently and a longer progression-free survival, but the numbers are undoubtedly too small for definite conclusions and our results have to be confirmed by other much larger studies.

In conclusion, the results of different preclinical studies indicate that the addition of a somatostatin analogue (with or without combination with an antiprolactin) may enhance the anti-tumour efficacy of anti-oestrogens in the treatment of breast cancer (Huynh et al, 1994; Weckbecker et al, 1994; Bogden et al, 1995). Our first randomized clinical study on triple therapy showed that in principle such an approach is clinically feasible and caused significant endocrine effects. A large multicentre randomized study in metastatic breast cancer, using a depot preparation of octreotide instead of daily injections, is warranted (and on-going) to prove the presence of such potential extra beneficial anti-tumour effect.

### ACKNOWLEDGEMENTS

We would like to thank the Dutch Cancer Society (grants RRTI 88–9 and IKR 90–18) and AG Sandoz for support, our colleagues JThP Janssen, P van Liessum, JJ Croles and C van der Heul for their contribution, and Miss F Smits and P Bos for typing the manuscript. Supported by grants RRTI 88–9 and IKR 90–18 of the Dutch Cancer Society, and by Sandoz AG.

### REFERENCES

Alexieva-Figusch J, Blankenstein MA, Hop WCJ, Klijn JGM, Lamberts SWJ, de Jong FH, Docter R, Adlercreutz H and van Gilse HA (1984) Treatment of metastatic breast cancer patients with different dosages of megestrol acetate; dose relations, metabolic and endocrine effects. *Eur J Cancer Clin Oncol* **20**: 33–40

Arteaga CL and Osborne CK (1989) Growth inhibition of human breast cancer cells in vitro with an antibody against the type I somatomedin receptor. *Cancer Res* **49**: 6237–6241

Bogden AE, Keyes SR, Grant W, Zwicker S, Silver M, Batista IL and Lepage D (1995) Therapeutic benefit of a lanreotide-tamoxifen combination in the treatment of breast tumors. *Proc Am Assoc Cancer Res* **36**:1507A

© Cancer Research Campaign 1998

Bonneterre J, Mauriac L, Weber B, Roche H, Fargeot P, Tubiana-Hulin M, Sevin M, Chollet P and Cappelaere P (1988) Tamoxifen plus bromocriptine versus tamoxifen plus placebo in advanced breast cancer: results of a double-blind multicentre clinical trial. *Eur J Cancer Clin Oncol* **24**: 1851–1853

Bonneterre J, Peyrat JP, Beuscart R and Demaille A (1990) Biological and clinical aspects of prolactin receptors (PRL-R) in human breast cancer. *J Steroid Biochem Molec Biol* **37**: 977–981

Buscail L, Estève JP, Saint-Laurent N, Bertrand V, Reisine T, O'Carroll AM, Bell GI, Schally AV, Vaysse N and Susini C (1995) Inhibition of cell proliferation by the somatostatin analogue RC-160 is mediated by somatostatin receptor subtypes SSTR2 and SSTR5 through different mechanisms. *Proc Natl Acad Sci* **92**: 1580–1584

Butta A, Maclennan K, Flanders KC, Sacks NPM, Smith I, McKinna A, Dowsett M, Wakefield LM, Sporn MB, Baum M and Colletta AA (1992) Induction of transforming growth factor β in human breast cancer in vivo following tamoxifen treatment. *Cancer Res* **52**: 4261–4264

Chiodera P, Volpi R, D'Amato L, Fatone M, Cigarini C, Fava A, Caiazza A, Rossi G and Coiro V (1986) Inhibition by somatostatin of LH-RH-induced LH release in normal menstruating women. *Gynaecol Obstet Invest* **22**: 17–21

Clarke R, Dickson RB and Lippman ME (1992) Hormonal aspects of breast cancer: growth factors, drugs and stromal interactions. *Crit Rev Oncol/Hematol* **12**: 1–23

Coletti RB, Roberts JD, Devlin JT and Copeland KC (1989) Effects of tamoxifen on plasma insulin-like growth factor I in patients with breast cancer. *Cancer Res* **49**: 1882–1884

Cullen KJ, Lippman ME, Chow D, Hill S, Rosen N and Zwiebel JA (1992) Insulin-like growth factor-II expression in MCF-7 cells induces phenotypic changes associated with malignant progression. *Mol Endocrinol* **6**: 91–100

Davoren JB and Hsueh AJW (1986) Growth hormone increases ovarian levels of immunoreactive somatomedin C/insulin-like growth factor I in vivo. *Endocrinol* **118**: 888–890

Dogliotti L, Robustelli Delle Cuna G, Di Carlo F and Brompa Italian Coop Group (1987) Medroxyprogesterone acetate high-dose (MPA-MD) versus MPA-HD plus bromocriptine in advanced breast cancer: preliminary results of a multicentre randomized clinical trial. In *Hormonal Manipulation of Cancer: Peptides, Growth Factors and New Anti (Steroidal) Agents. EORTC Monograph Series* **18** Klijn, JGM and Paridaens R and Foekens JA (eds), pp. 183–193. New York: Raven Press

Emerman JT, Leaky M, Gout PW and Bruchovsky N (1985) Elevated growth hormone levels in sera from breast cancer patients. *Horm Metab Res* **17**: 421–424

Engelsman E, Heuson JC, Blonk-Van Der Wijst J, Drochmans A, Maass H, Cheix F, Sobrinho LG and Nowakowski N (1975) Controlled clinical trials of L-dopa and nafoxidine in advanced breast cancer: an EORTC study. *Br Med J* **2**: 714–715

European Breast Cancer Group (1972) Clinical trial of 2-Br-α-ergocryptine (CB-154) in advanced breast cancer. *Eur J Cancer* **8**: 155–156

van Eijck CHJ, Krenning EP, Bootsma A, Oei HY, Van Pel R, Lindemans J, Jeekel J, Reubi JC and Lamberts SWJ (1994) Somatostatin-receptor scintigraphy in primary breast cancer. *Lancet* **343**: 640–643

Fekete M, Wittliff JL and Schally AV (1989) Characteristics and distribution of receptors for [D-Trp$^6$]-luteinizing-hormone-releasing hormone, somatostatin, epidermal growth factor, and sex steroids in 500 biopsy samples of human breast cancer. *J Clin Lab Analysis* **3**: 137–141

Fentimen IS, Brame K, Chaudary MA, Champlejohn RS, Wang DY and Millis RR (1988) Perioperative bromocriptine adjuvant treatment for operable breast cancer. *Lancet* **1**: 609–610

Fields K, Kulig E and Lloyd RV (1993) Detection of prolactin messenger RNA in mammary and other normal and neoplastic tissues by polymerase chain reaction. *Lab Invest* **68**: 354–360

Foekens JA, Portengen H, Van Putten WLJ, Reubi JC, Trapman AMAC, Alexieva-Figusch J and Klijn JGM (1989*a*) The prognostic significance of receptors for insulin-like growth factor-I, somatostatin, and epidermal growth factor in human primary breast cancer. *Cancer Res* **49**: 7002–7009

Foekens JA, Portengen H, Janssen M and Klijn JGM (1989*b*) Insulin-like growth factor-I-receptors and insulin-like growth factor-I-like activity in human primary breast cancer. *Cancer* **63**: 2139–2147

Freiss G, Rochefort H and Vignon F (1990) Mechanisms of 4-hydroxytamoxifen anti-growth factor activity in breast cancer cells: Alteration of growth factor receptor binding sites and tyrosine kinase activity. *Biochem Biophys Res Commun* **173**: 919–926

Ginsburg E and Vonderhaar BK (1995) Prolactin synthesis and secretion by human breast cancer cells. *Cancer Res* **55**: 2591–2595

Grisoli F, Vincentelli F, Foa J, Lavail G and Salamon G (1981) Effect of bromocriptine on brain metastasis in breast cancer. *Lancet* **2**: 745–746

Holtkamp W, Nagel GE, Wander HE, Rauschecker HF and Von Heyden D (1984) Hyperprolactinemia is an indicator of progressive disease and poor prognosis in advanced breast cancer. *Int J Cancer* **34**: 323–328

Holtkamp W and Nagel GA (1990) Remission of metastatic breast cancer after combined somatostatin and antiprolactin treatment. *Eur J Cancer* **26**: 177

Huynh H and Pollak M (1994) Enhancement of tamoxifen-induced suppression of insulin-like growth factor I gene expression and serum level by a somatostatin analogue. *Biochem Biophys Res Commun* **203**: 253–259

Kelly PA, Djiane J, Postel-Vinay MC and Edery M (1991) The prolactin/growth hormone receptor family. *Endocrine Rev* **12**: 235–251

Kiang DT, Kollander R, Kiang B and Kao PC (1992) Role of plasma IGF-I in endocrine therapy for breast cancer (abstract 31). *Proc Am Soc Clin Oncol* **11**: 51

Klijn JGM, De Jong FH, Lamberts SWJ and Blankenstein MA (1985) LHRH-agonist treatment in clinical and experimental human breast cancer. *J Steroid Biochem* **23**: 867–878

Klijn JGM, Hoff AM, Planting ASTH, Verweij J, Kok TC, Lamberts SWJ, Portengen H and Foekens JA (1990*a*) Treatment of patients with metastatic pancreatic and gastrointestinal tumors with the somatostatin analog Sandostatin: A phase II study including endocrine effects. *Br J Cancer* **62**: 627–630

Klijn JGM, Setyono-Han B, Bakker GH, Van Der Burg MEL, Bontenbal M, Peters HA, Sieuwerts AM, Berns PMJJ and Foekens JA (1990*b*) Growth factor-receptor pathway interfering treatment by somatostatin analogs and suramin: preclinical and clinical studies. *J Steroid Biochem Molec Biol* **37**: 1089–1095

Klijn JGM, Berns PMJJ, Bontenbal M, Alexieva-Figusch J and Foekens JA (1992) Clinical breast cancer, new developments in selection and endocrine treatment of patients. *J Steroid Biochem Molec Biol* **43**: 211–221

Klijn JGM, Berns PMJJ and Foekens JA (1993) Prognostic factors and response to therapy in breast cancer. *Cancer Surv* **18**: 165–198

Kopp A, Jonat W, Schmall M and Knabbe C (1995) Transforming growth factor beta 2 (TGF-beta 2) levels in plasma of patients with metastatic breast cancer treated with tamoxifen. *Cancer Res* **55**: 4512–4515

Lamberts SWJ and Macleod RM (1990) Regulation of prolactin secretion at the level of the lactotroph. *Physiolog Rev* **70**: 279–318

Lamberts SWJ, Krenning EP and Reubi JC (1991) The role of somatostatin and its analogs in the diagnosis and treatment of tumors. *Endocrine Rev* **12**: 450–482

Lamberts SWJ, Van Der Lely AJ, De Herder WW and Hofland LJ (1996) Octreotide. *N Engl J Med* **334**: 246–254

Lönning PE, Hall K, Aakvaag A and Lien EA (1992) Influence of tamoxifen on plasma levels of insulin-like growth factor I and insulin-like growth factor binding protein I in breast cancer patients. *Cancer Res* **52**: 4719–4723

Malaab SA, Pollak MN and Goodyer CG (1992) Direct effects of tamoxifen on growth hormone secretion by pituitary cells *in vitro*. *Eur J Cancer* **28A**: 788–793

Malarkey WB, Kennedy M, Allred LE and Milo G (1983) Physiological concentrations of prolactin can promote the growth of human breast tumor cells in culture. *J Clin Endocrinol Metab* **56**: 673–677

Manni A, Wright C, Davis G, Glenn J, Joehl R and Feil P (1986) Promotion by prolactin of the growth of human breast neoplasms cultured in vitro in the soft agar clonogenic assay. *Cancer Res* **46**: 1668–1672

Manni A, Boucher AE, Demers LM, Harvey HA, Lipton A, Simmonds MA and Bartholomew M (1989) Endocrine effects of combined somatostatin analog and bromocriptine therapy in women with advanced breast cancer. *Breast Cancer Res Treat* **14**: 289–298

Mershon J, Sall W, Mitchner N and Ben-Jonathan N (1995) Prolactin is a local growth factor in rat mammary tumors. *Endocrinology* **136**: 3619–3623

Minton JP (1974) The response of breast cancer patients with bone pain to L-dopa. *Cancer* **33**: 358–363

Mol JA, Van Garderen E, Selman PJ, Wolfswinkel J, Rijnberk A and Rutteman GR (1995) Growth hormone mRNA in mammary gland tumors of dogs and cats. *J Clin Invest* **95**: 2028–2034

Morten H, Howell A, Shalet SM, Robinson L and Anderson E (1988) Measurement of immunoreactive and bioactive lactogenic hormones in advanced breast cancer patients treated with bromocriptine and SMS 201–995. *J Endocrinol* **119S**

Murphy LJ, Vrhovsek E, Sutherland RL and Lazarus L (1984) Growth hormone binding to cultured human breast cancer cells. *J Clin Endocrinol Metab* **58**: 149–156

Osborne CK, Clemmons DR and Arteaga CL (1990) Regulation of breast cancer growth by insulin-like growth factors. *J Steroid Biochem Molec Biol* **37**: 805–809

© Cancer Research Campaign 1998

Pekonen F, Partanen S, Mäkinen T and Rutanen EM (1988) Receptors for epidermal growth factor and insulin-like growth factor-I and their relation to steroid receptors in human breast cancer. *Cancer Res* **48**: 1343–1347

Peyrat JP, Bonneterre J, Beuscart B, Djiane J and Demaille A (1988*a*) Insulin-like growth factor-I receptors in human breast cancer and their relation to estradiol and progesterone receptors. *Cancer Res* **48**: 6429–6433

Peyrat JP, Bonneterre J, Laurent JC, Louchez MM, Amrani S, Leroy-Martin B, Vilain MO, Delobelle A and Demaille A (1988*b*) Presence and characterisation of insulin-like growth factor-I receptors in benign breast diseases. *Eur J Cancer Clin Oncol* **24**: 1425–1431

Peyrat JP, Bonneterre J, Hecquet B, Vennin P, Louchez MM, Fournier C, Lefebvre J and Demaille A (1993) Plasma insulin-like growth factor-I (IGF-I) concentrations in human breast cancer. *Eur J Cancer* **29A**: 492–497

Pollak MN, Polychronakos C and Yousefi S (1988) Characterization of insulin-like growth factor I (IGF-I) receptors of human breast cancer cells. *Biochem Biophys Res Commun* **154**: 326–331

Pollak MN, Polychronakos C and Guyda H (1989) Somatostatin analogue SMS 201–995 reduces serum IGF-I levels in patients with neoplasms potentially dependent on IGF-I. *Anticancer Res* **9**: 889–891

Pollak M, Costantino J, Polychronakos C, Blauer SA, Guyda H, Redmond C, Fisher B and Margolese R (1990) Effect of tamoxifen on serum insulin-like growth factor I levels in stage I breast cancer patients. *J Natl Cancer Inst* **82**: 1693–1697

Pollak MN, Huynh T, Pratt H and Lefebvre S (1992) Tamoxifen reduces serum insulin-like growth factor I (IGF-I). *Breast Cancer Res Treat* **22**: 91–100

Pratt SE and Pollak MN (1993) Estrogen and antiestrogen modulation of MCF7 human breast cancer cell proliferation is associated with specific alterations in accumulation of insulin-like growth factor-binding proteins in conditioned media. *Cancer Res* **53**: 5193–5198

Prévost G, Hosford D and Thomas F (1994) Receptors for somatostatin and somatostatin analogues in human breast tumors. *Ann NY Acad Sci* **133**: 147–154

Rasmussen C, Bergh T, Wide L and Brownell J (1987) CV 205-502: a new long-acting drug for inhibition of prolactin hypersecretion. *Clin Endocrinol* **26**: 321–326

Reed MJ, Christodoulides A, Koistinen R, Seppälä M, Teale JB and Ghilchik MW (1992) The effect of endocrine therapy with medroxyprogesterone acetate, 4-hydroxyandrostenedione or tamoxifen on plasma concentrations of insulin-like growth factor (IGF)-I, IGF-II and IGFBP-I in women with advanced breast cancer. *Int J Cancer* **52**: 208–212

Reubi JC, Waser B, Foekens JA, Klijn JGM, Lamberts SWJ and Laissue J (1990) Somatostatin receptor incidence and distribution in breast cancer using receptor autoradiography. Relationship to EGF receptors. *Int J Cancer* **46**: 416–420

Van Roozendaal CEP, Klijn JGM, Van Ooijen B, Claassen C, Eggermont AMM, Henzen-Logmans SC and Foekens JA (1995) Transforming growth factor beta secretion from primary breast cancer fibroblasts. *Mol Cell Endocrinol* **111**: 1–6

Rose DP, Gottardis M and Noonan JJ (1983) Rat mammary carcinoma regressions during suppression of serum growth hormone and prolactin. *Anticancer Res* **3**: 323

Santen RJ, Manni A, Harvey H and Redmond C (1990) Endocrine treatment of breast cancer in women. *Endocrine Rev* **11**: 221–265

Schally AV, Redding TW, Cai RZ, Paz JI, Ben-David M and Comaru-Schally AM (1987) Somatostatin analogs in the treatment of various experimental tumors. In *Hormonal Manipulation of Cancer: Peptides, Growth Factors and new Anti (steroidal) Agents*. Monograph Series of the EORTC, Vol. 18, Klijn JGM, Paridaens R and Foekens JA (eds), pp. 431–441. New York: Raven Press

Schally AV (1988) Oncological applications of somatostatin analogues. *Cancer Res* **48**: 6977–6985

Setyono-Han B, Henkelman MS, Foekens JA and Klijn JGM (1987) Direct inhibitory effects of somatostatin (analogues) on the growth of human breast cancer cells. *Cancer Res* **47**: 1566–1570

Srkalovic G, Cai RZ and Schally AV (1990) Evaluation of receptors for somatostatin in various tumors using different analogs. *J Clin Endocrinol Metab* **70**: 661–669

Szende B, Lapis K, Redding TW, Srkalovic G and Schally AV (1989) Growth inhibition of MXT mammary carcinoma by enhancing programmed cell death (apoptosis) with analogs of LH-RH and somatostatin. *Breast Cancer Res Treat* **14**: 307–314

Tannenbaum SG, Gurd W, Lapointe M and Pollak M (1992) Tamoxifen attenuates pulsatile growth hormone secretion: mediation in part by somatostatin. *Endocrinology* **130**: 3395–3401

Vennin P, Peyrat JP, Bonneterre J, Louchez MM, Harris AG and Demaille A (1989) Effect of the long-acting somatostatin analog SMS 201–995 (Sandostatin) in advanced breast cancer. *Anticancer Res* **9**: 153–156

Weber C, Merriam L, Kotschitzky T, Kar PF, Benson M, Forde K and Logerfo P (1989) Inhibition of growth of human breast carcinomas *in vivo* by somatostatin analog SMS 201–995: treatment of nude mouse xenografts. *Surgery* **106**: 416–422

Weckbecker G, Liu R, Tolcsvai L and Bruns C (1992) Antiproliferative effects of the somatostatin analogue octreotide (SMS 201–995) on ZR-75-1 human breast cancer cells *in vivo* and *in vitro*. *Cancer Res* **52**: 4973–4978

Weckbecker G, Tolcsvai L, Stolz B, Pollak M and Bruns C (1994) Somatostatin analogue octreotide enhances the antineoplastic effects of tamoxifen and ovariectomy on 7,12-Dimethylbenz(α)anthracene-induced rat mammary carcinomas. *Cancer Res* **54**: 6334–6337

Winston R, Kao PC and Kiang DT (1994) Regulation of insulin-like growth factors by antiestrogen. *Breast Cancer Res Treat* **31**: 107–115

© Cancer Research Campaign 1998


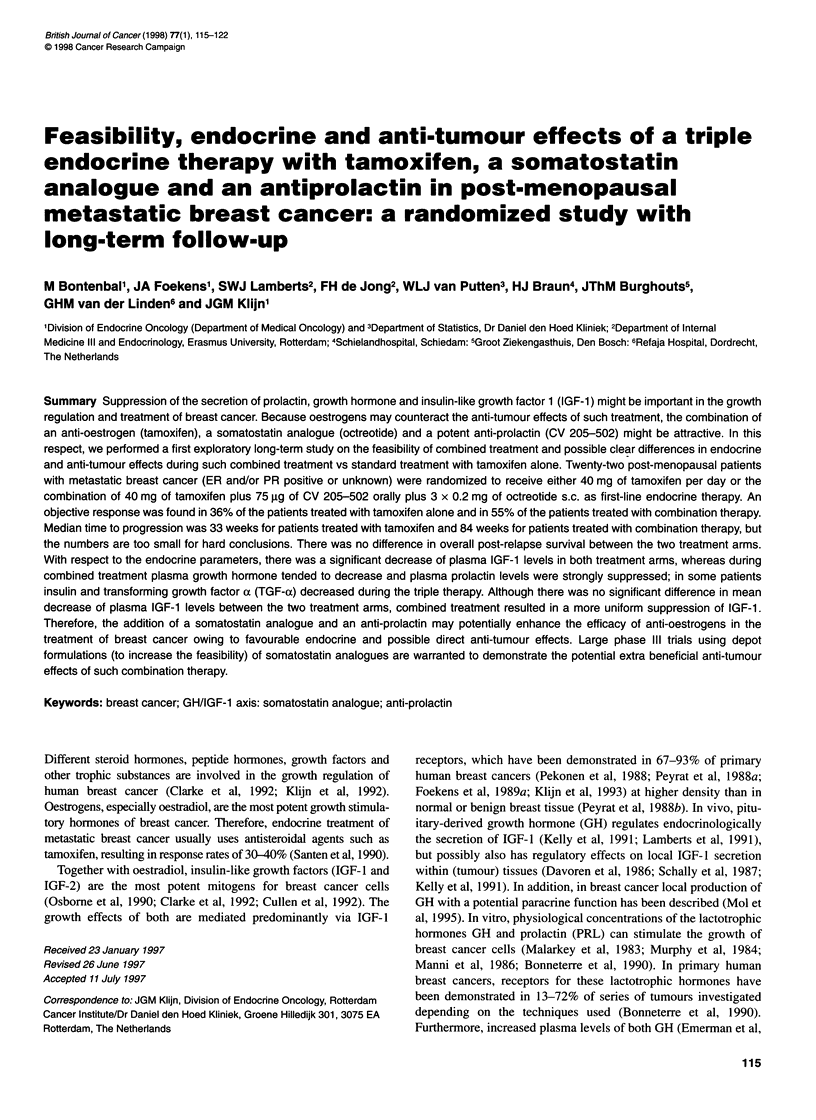

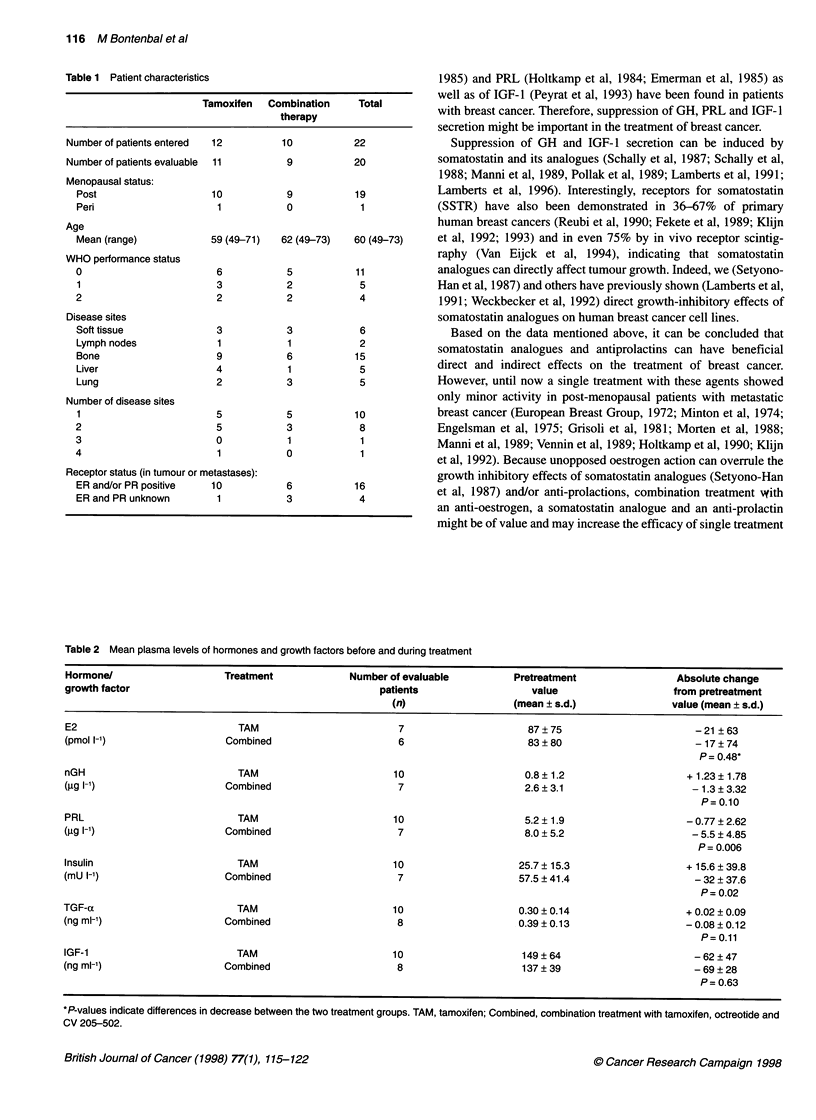

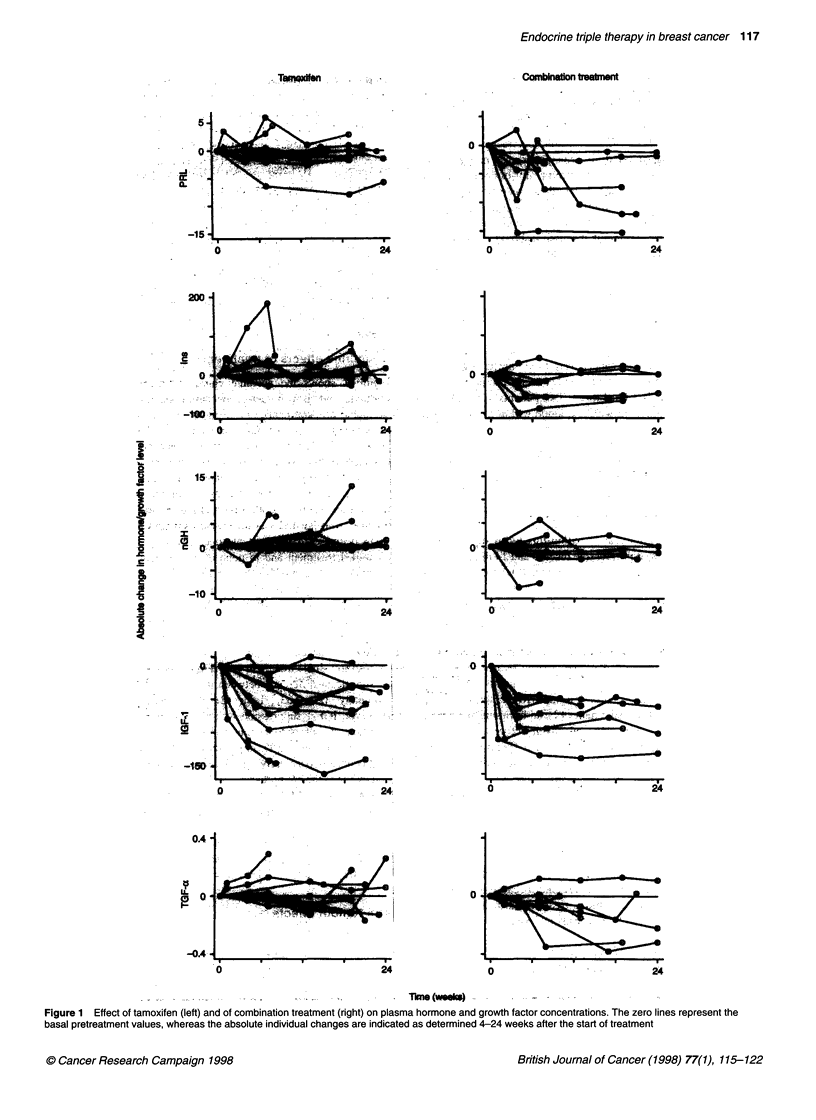

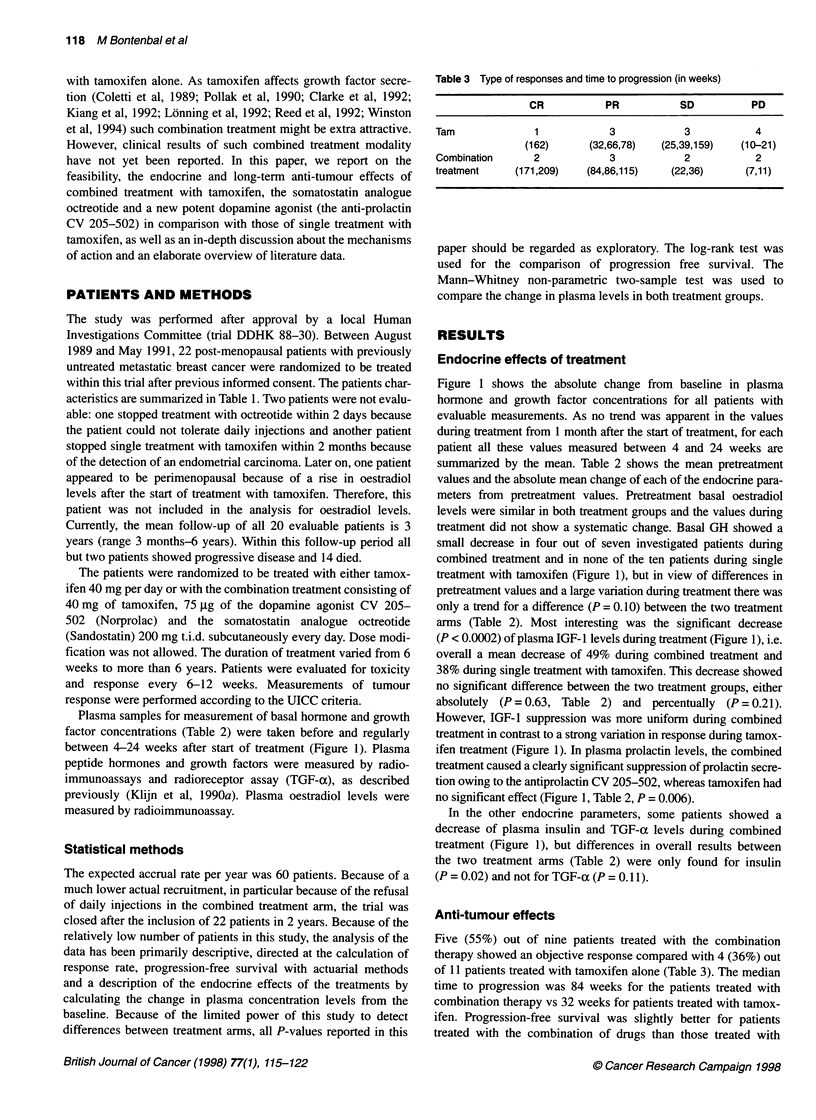

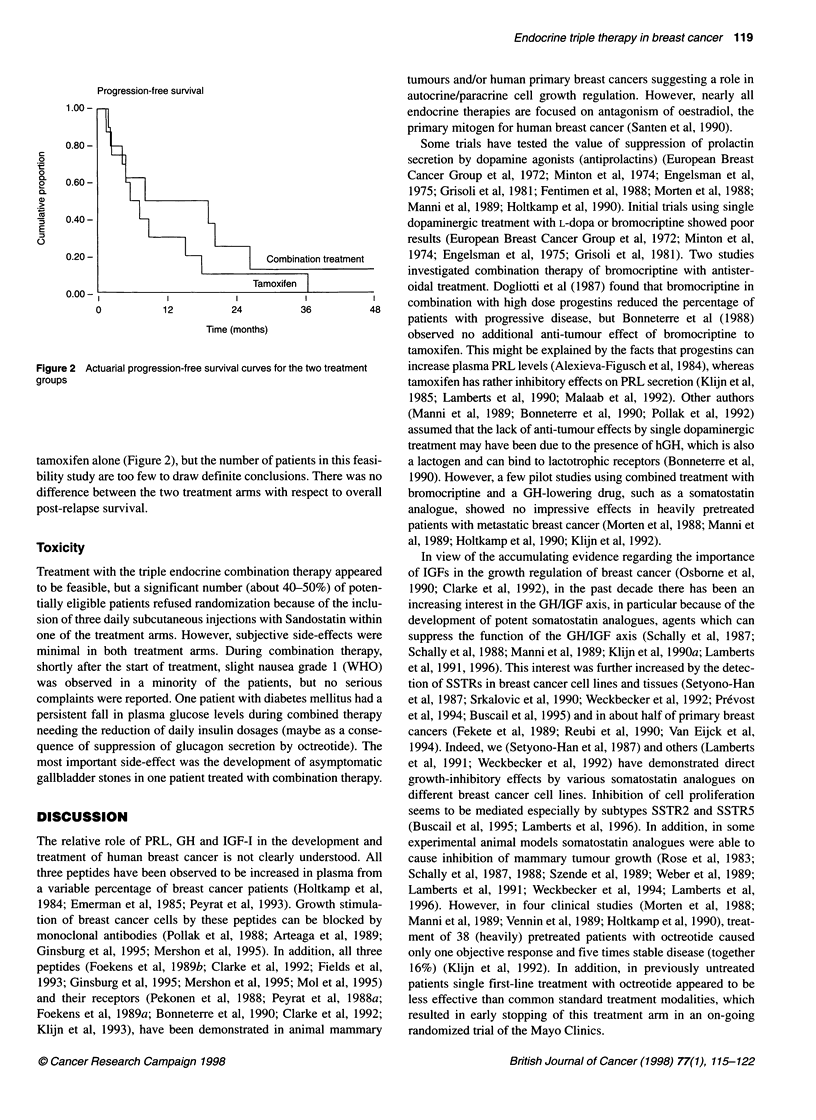

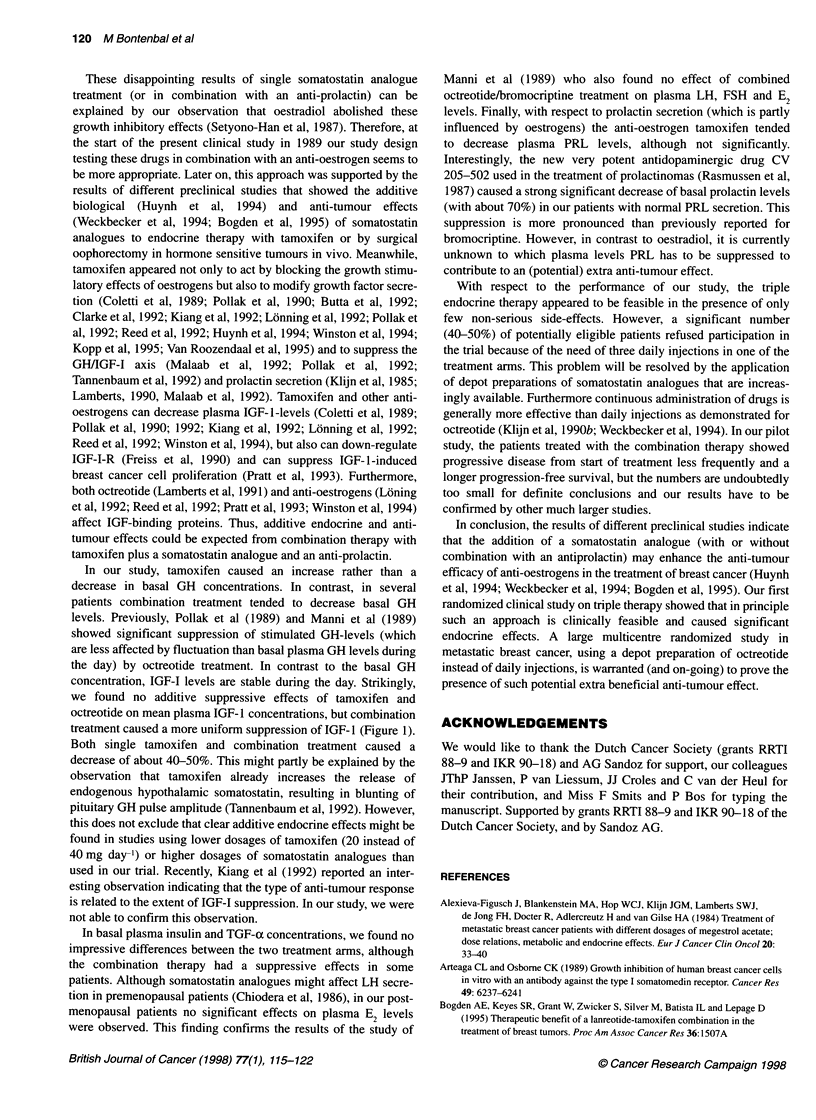

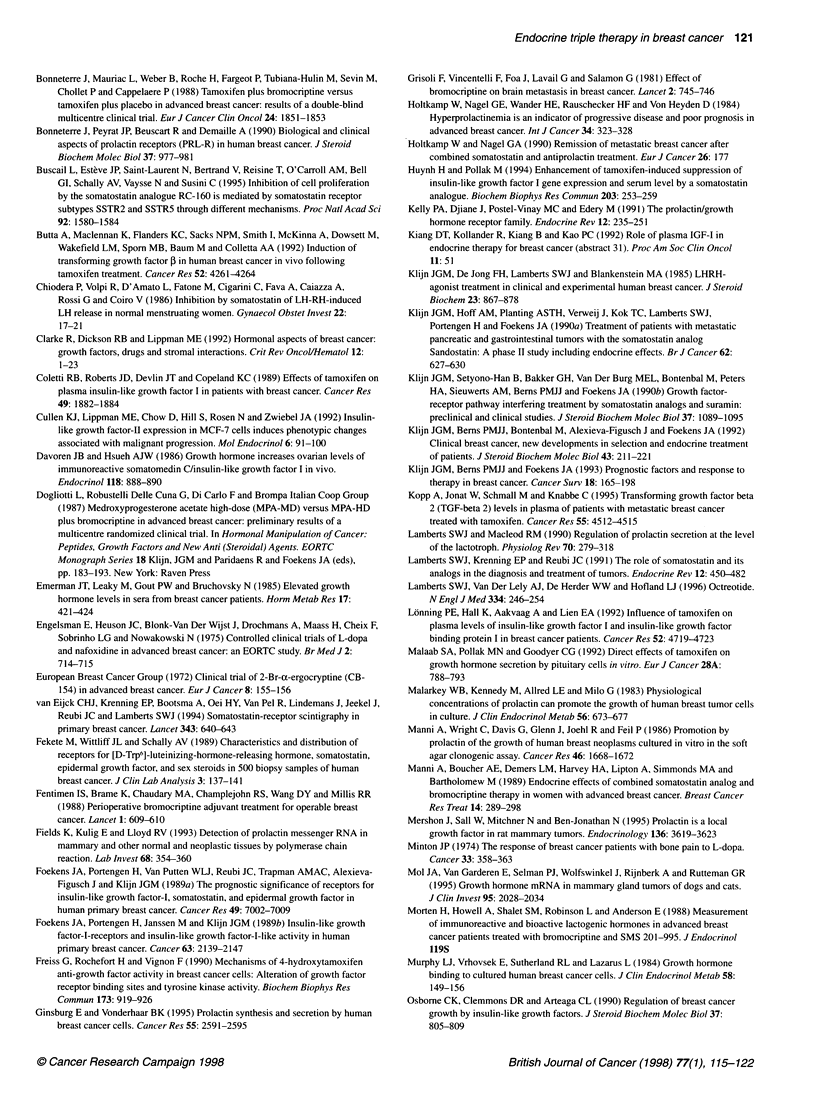

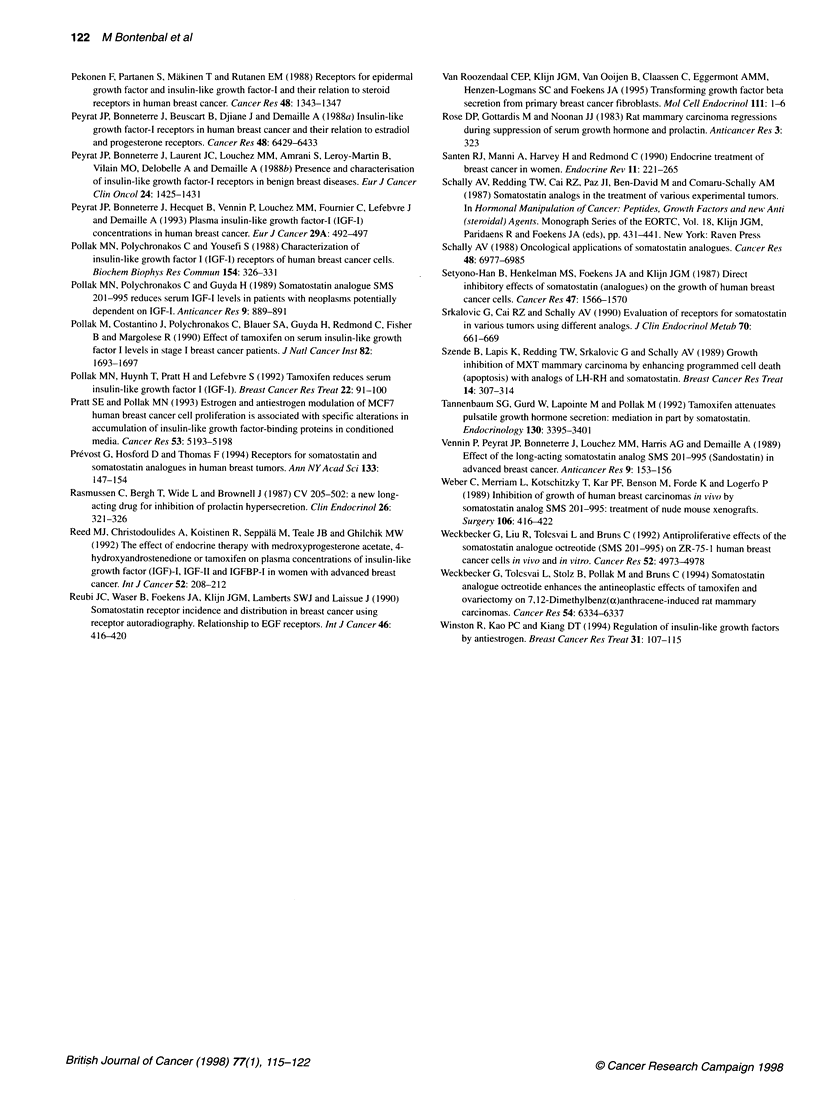


## References

[OCR_00707] Alexieva-Figusch J., Blankenstein M. A., Hop W. C., Klijn J. G., Lamberts S. W., de Jong F. H., Docter R., Adlercreutz H., van Gilse H. A. (1984). Treatment of metastatic breast cancer patients with different dosages of megestrol acetate; dose relations, metabolic and endocrine effects.. Eur J Cancer Clin Oncol.

[OCR_00714] Arteaga C. L., Osborne C. K. (1989). Growth inhibition of human breast cancer cells in vitro with an antibody against the type I somatomedin receptor.. Cancer Res.

[OCR_00728] Bonneterre J., Mauriac L., Weber B., Roche H., Fargeot P., Tubiana-Hulin M., Sevin M., Chollet P., Cappelaere P. (1988). Tamoxifen plus bromocriptine versus tamoxifen plus placebo in advanced breast cancer: results of a double blind multicentre clinical trial.. Eur J Cancer Clin Oncol.

[OCR_00735] Bonneterre J., Peyrat J. P., Beuscart R., Demaille A. (1990). Biological and clinical aspects of prolactin receptors (PRL-R) in human breast cancer.. J Steroid Biochem Mol Biol.

[OCR_00740] Buscail L., Estève J. P., Saint-Laurent N., Bertrand V., Reisine T., O'Carroll A. M., Bell G. I., Schally A. V., Vaysse N., Susini C. (1995). Inhibition of cell proliferation by the somatostatin analogue RC-160 is mediated by somatostatin receptor subtypes SSTR2 and SSTR5 through different mechanisms.. Proc Natl Acad Sci U S A.

[OCR_00748] Butta A., MacLennan K., Flanders K. C., Sacks N. P., Smith I., McKinna A., Dowsett M., Wakefield L. M., Sporn M. B., Baum M. (1992). Induction of transforming growth factor beta 1 in human breast cancer in vivo following tamoxifen treatment.. Cancer Res.

[OCR_00754] Chiodera P., Volpi R., d'Amato L., Fatone M., Cigarini C., Fava A., Caiazza A., Rossi G., Coiro V. (1986). Inhibition by somatostatin of LH-RH-induced LH release in normal menstruating women.. Gynecol Obstet Invest.

[OCR_00760] Clarke R., Dickson R. B., Lippman M. E. (1992). Hormonal aspects of breast cancer. Growth factors, drugs and stromal interactions.. Crit Rev Oncol Hematol.

[OCR_00765] Colletti R. B., Roberts J. D., Devlin J. T., Copeland K. C. (1989). Effect of tamoxifen on plasma insulin-like growth factor I in patients with breast cancer.. Cancer Res.

[OCR_00770] Cullen K. J., Lippman M. E., Chow D., Hill S., Rosen N., Zwiebel J. A. (1992). Insulin-like growth factor-II overexpression in MCF-7 cells induces phenotypic changes associated with malignant progression.. Mol Endocrinol.

[OCR_00775] Davoren J. B., Hsueh A. J. (1986). Growth hormone increases ovarian levels of immunoreactive somatomedin C/insulin-like growth factor I in vivo.. Endocrinology.

[OCR_00791] Emerman J. T., Leahy M., Gout P. W., Bruchovsky N. (1985). Elevated growth hormone levels in sera from breast cancer patients.. Horm Metab Res.

[OCR_00796] Engelsman E., Heuson J. C., Blonk Van Der Wijst J., Drochmans A., Maass H., Cheix F., Sobrinho L. G., Nowakowski H. (1975). Controlled clinical trial of L-dopa and nafoxidine in advanced breast cancer: an E.O.R.T.C. study.. Br Med J.

[OCR_00811] Fekete M., Wittliff J. L., Schally A. V. (1989). Characteristics and distribution of receptors for [D-TRP6]-luteinizing hormone-releasing hormone, somatostatin, epidermal growth factor, and sex steroids in 500 biopsy samples of human breast cancer.. J Clin Lab Anal.

[OCR_00817] Fentiman I. S., Brame K., Chaudary M. A., Camplejohn R. S., Wang D. Y., Millis R. R. (1988). Perioperative bromocriptine adjuvant treatment for operable breast cancer.. Lancet.

[OCR_00822] Fields K., Kulig E., Lloyd R. V. (1993). Detection of prolactin messenger RNA in mammary and other normal and neoplastic tissues by polymerase chain reaction.. Lab Invest.

[OCR_00829] Foekens J. A., Portengen H., van Putten W. L., Trapman A. M., Reubi J. C., Alexieva-Figusch J., Klijn J. G. (1989). Prognostic value of receptors for insulin-like growth factor 1, somatostatin, and epidermal growth factor in human breast cancer.. Cancer Res.

[OCR_00838] Freiss G., Rochefort H., Vignon F. (1990). Mechanisms of 4-hydroxytamoxifen anti-growth factor activity in breast cancer cells: alterations of growth factor receptor binding sites and tyrosine kinase activity.. Biochem Biophys Res Commun.

[OCR_00844] Ginsburg E., Vonderhaar B. K. (1995). Prolactin synthesis and secretion by human breast cancer cells.. Cancer Res.

[OCR_00848] Grisoli F., Vincentelli F., Foa J., Lavail G., Salamon G. (1981). Effect of bromocriptine on brain metastasis in breast cancer.. Lancet.

[OCR_00852] Holtkamp W., Nagel G. A., Wander H. E., Rauschecker H. F., von Heyden D. (1984). Hyperprolactinemia is an indicator of progressive disease and poor prognosis in advanced breast cancer.. Int J Cancer.

[OCR_00861] Huynh H., Pollak M. (1994). Enhancement of tamoxifen-induced suppression of insulin-like growth factor I gene expression and serum level by a somatostatin analogue.. Biochem Biophys Res Commun.

[OCR_00866] Kelly P. A., Djiane J., Postel-Vinay M. C., Edery M. (1991). The prolactin/growth hormone receptor family.. Endocr Rev.

[OCR_00899] Klijn J. G., Berns E. M., Foekens J. A. (1993). Prognostic factors and response to therapy in breast cancer.. Cancer Surv.

[OCR_00894] Klijn J. G., Berns P. M., Bontenbal M., Alexieva-Figusch J., Foekens J. A. (1992). Clinical breast cancer, new developments in selection and endocrine treatment of patients.. J Steroid Biochem Mol Biol.

[OCR_00880] Klijn J. G., Hoff A. M., Planting A. S., Verweij J., Kok T., Lamberts S. W., Portengen H., Foekens J. A. (1990). Treatment of patients with metastatic pancreatic and gastrointestinal tumours with the somatostatin analogue Sandostatin: a phase II study including endocrine effects.. Br J Cancer.

[OCR_00888] Klijn J. G., Setyono-Han B., Bakker G. H., van der Burg M. E., Bontenbal M., Peters H. A., Sieuwerts A. M., Berns P. M., Foekens J. A. (1990). Growth factor-receptor pathway interfering treatment by somatostatin analogs and suramin: preclinical and clinical studies.. J Steroid Biochem Mol Biol.

[OCR_00875] Klijn J. G., de Jong F. H., Lamberts S. W., Blankenstein M. A. (1985). LHRH-agonist treatment in clinical and experimental human breast cancer.. J Steroid Biochem.

[OCR_00903] Kopp A., Jonat W., Schmahl M., Knabbe C. (1995). Transforming growth factor beta 2 (TGF-beta 2) levels in plasma of patients with metastatic breast cancer treated with tamoxifen.. Cancer Res.

[OCR_00912] Lamberts S. W., Krenning E. P., Reubi J. C. (1991). The role of somatostatin and its analogs in the diagnosis and treatment of tumors.. Endocr Rev.

[OCR_00908] Lamberts S. W., Macleod R. M. (1990). Regulation of prolactin secretion at the level of the lactotroph.. Physiol Rev.

[OCR_00916] Lamberts S. W., van der Lely A. J., de Herder W. W., Hofland L. J. (1996). Octreotide.. N Engl J Med.

[OCR_00920] Lønning P. E., Hall K., Aakvaag A., Lien E. A. (1992). Influence of tamoxifen on plasma levels of insulin-like growth factor I and insulin-like growth factor binding protein I in breast cancer patients.. Cancer Res.

[OCR_00925] Malaab S. A., Pollak M. N., Goodyer C. G. (1992). Direct effects of tamoxifen on growth hormone secretion by pituitary cells in vitro.. Eur J Cancer.

[OCR_00930] Malarkey W. B., Kennedy M., Allred L. E., Milo G. (1983). Physiological concentrations of prolactin can promote the growth of human breast tumor cells in culture.. J Clin Endocrinol Metab.

[OCR_00940] Manni A., Boucher A. E., Demers L. M., Harvey H. A., Lipton A., Simmonds M. A., Bartholomew M. (1989). Endocrine effects of combined somatostatin analog and bromocriptine therapy in women with advanced breast cancer.. Breast Cancer Res Treat.

[OCR_00935] Manni A., Wright C., Davis G., Glenn J., Joehl R., Feil P. (1986). Promotion by prolactin of the growth of human breast neoplasms cultured in vitro in the soft agar clonogenic assay.. Cancer Res.

[OCR_00946] Mershon J., Sall W., Mitchner N., Ben-Jonathan N. (1995). Prolactin is a local growth factor in rat mammary tumors.. Endocrinology.

[OCR_00950] Minton J. P. (1974). Proceedings: The response of breast cancer patients with bone pain to L-dopa.. Cancer.

[OCR_00954] Mol J. A., van Garderen E., Selman P. J., Wolfswinkel J., Rijinberk A., Rutteman G. R. (1995). Growth hormone mRNA in mammary gland tumors of dogs and cats.. J Clin Invest.

[OCR_00966] Murphy L. J., Vrhovsek E., Sutherland R. L., Lazarus L. (1984). Growth hormone binding to cultured human breast cancer cells.. J Clin Endocrinol Metab.

[OCR_00971] Osborne C. K., Clemmons D. R., Arteaga C. L. (1990). Regulation of breast cancer growth by insulin-like growth factors.. J Steroid Biochem Mol Biol.

[OCR_00980] Pekonen F., Partanen S., Mäkinen T., Rutanen E. M. (1988). Receptors for epidermal growth factor and insulin-like growth factor I and their relation to steroid receptors in human breast cancer.. Cancer Res.

[OCR_00985] Peyrat J. P., Bonneterre J., Beuscart R., Djiane J., Demaille A. (1988). Insulin-like growth factor 1 receptors in human breast cancer and their relation to estradiol and progesterone receptors.. Cancer Res.

[OCR_00996] Peyrat J. P., Bonneterre J., Hecquet B., Vennin P., Louchez M. M., Fournier C., Lefebvre J., Demaille A. (1993). Plasma insulin-like growth factor-1 (IGF-1) concentrations in human breast cancer.. Eur J Cancer.

[OCR_00990] Peyrat J. P., Bonneterre J., Laurent J. C., Louchez M. M., Amrani S., Leroy-Martin B., Vilain M. O., Delobelle A., Demaille A. (1988). Presence and characterization of insulin-like growth factor 1 receptors in human benign breast disease.. Eur J Cancer Clin Oncol.

[OCR_01017] Pollak M. N., Huynh H. T., Lefebvre S. P. (1992). Tamoxifen reduces serum insulin-like growth factor I (IGF-I).. Breast Cancer Res Treat.

[OCR_01006] Pollak M. N., Polychronakos C., Guyda H. (1989). Somatostatin analogue SMS 201-995 reduces serum IGF-I levels in patients with neoplasms potentially dependent on IGF-I.. Anticancer Res.

[OCR_01001] Pollak M. N., Polychronakos C., Yousefi S., Richard M. (1988). Characterization of insulin-like growth factor I (IGF-I) receptors of human breast cancer cells.. Biochem Biophys Res Commun.

[OCR_01011] Pollak M., Costantino J., Polychronakos C., Blauer S. A., Guyda H., Redmond C., Fisher B., Margolese R. (1990). Effect of tamoxifen on serum insulinlike growth factor I levels in stage I breast cancer patients.. J Natl Cancer Inst.

[OCR_01021] Pratt S. E., Pollak M. N. (1993). Estrogen and antiestrogen modulation of MCF7 human breast cancer cell proliferation is associated with specific alterations in accumulation of insulin-like growth factor-binding proteins in conditioned media.. Cancer Res.

[OCR_01027] Prévost G., Hosford D., Thomas F. (1994). Receptors for somatostatin and somatostatin analogues in human breast tumors.. Ann N Y Acad Sci.

[OCR_01032] Rasmussen C., Bergh T., Wide L., Brownell J. (1987). CV 205-502: a new long-acting drug for inhibition of prolactin hypersecretion.. Clin Endocrinol (Oxf).

[OCR_01037] Reed M. J., Christodoulides A., Koistinen R., Seppälä M., Teale J. D., Ghilchik M. W. (1992). The effect of endocrine therapy with medroxyprogesterone acetate, 4-hydroxyandrostenedione or tamoxifen on plasma concentrations of insulin-like growth factor (IGF)-I, IGF-II and IGFBP-1 in women with advanced breast cancer.. Int J Cancer.

[OCR_01045] Reubi J. C., Waser B., Foekens J. A., Klijn J. G., Lamberts S. W., Laissue J. (1990). Somatostatin receptor incidence and distribution in breast cancer using receptor autoradiography: relationship to EGF receptors.. Int J Cancer.

[OCR_01056] Rose D. P., Gottardis M., Noonan J. J. (1983). Rat mammary carcinoma regressions during suppression of serum growth hormone and prolactin.. Anticancer Res.

[OCR_01061] Santen R. J., Manni A., Harvey H., Redmond C. (1990). Endocrine treatment of breast cancer in women.. Endocr Rev.

[OCR_01073] Schally A. V. (1988). Oncological applications of somatostatin analogues.. Cancer Res.

[OCR_01077] Setyono-Han B., Henkelman M. S., Foekens J. A., Klijn G. M. (1987). Direct inhibitory effects of somatostatin (analogues) on the growth of human breast cancer cells.. Cancer Res.

[OCR_01082] Srkalovic G., Cai R. Z., Schally A. V. (1990). Evaluation of receptors for somatostatin in various tumors using different analogs.. J Clin Endocrinol Metab.

[OCR_01087] Szende B., Lapis K., Redding T. W., Srkalovic G., Schally A. V. (1989). Growth inhibition of MXT mammary carcinoma by enhancing programmed cell death (apoptosis) with analogs of LH-RH and somatostatin.. Breast Cancer Res Treat.

[OCR_01093] Tannenbaum G. S., Gurd W., Lapointe M., Pollak M. (1992). Tamoxifen attenuates pulsatile growth hormone secretion: mediation in part by somatostatin.. Endocrinology.

[OCR_01098] Vennin P., Peyrat J. P., Bonneterre J., Louchez M. M., Harris A. G., Demaille A. (1989). Effect of the long-acting somatostatin analogue SMS 201-995 (Sandostatin) in advanced breast cancer.. Anticancer Res.

[OCR_01103] Weber C., Merriam L., Koschitzky T., Karp F., Benson M., Forde K., LoGerfo P. (1989). Inhibition of growth of human breast carcinomas in vivo by somatostatin analog SMS 201-995: treatment of nude mouse xenografts.. Surgery.

[OCR_01110] Weckbecker G., Liu R., Tolcsvai L., Bruns C. (1992). Antiproliferative effects of the somatostatin analogue octreotide (SMS 201-995) on ZR-75-1 human breast cancer cells in vivo and in vitro.. Cancer Res.

[OCR_01115] Weckbecker G., Tolcsvai L., Stolz B., Pollak M., Bruns C. (1994). Somatostatin analogue octreotide enhances the antineoplastic effects of tamoxifen and ovariectomy on 7,12-dimethylbenz(alpha)anthracene-induced rat mammary carcinomas.. Cancer Res.

[OCR_01121] Winston R., Kao P. C., Kiang D. T. (1994). Regulation of insulin-like growth factors by antiestrogen.. Breast Cancer Res Treat.

[OCR_00806] van Eijck C. H., Krenning E. P., Bootsma A., Oei H. Y., van Pel R., Lindemans J., Jeekel J., Reubi J. C., Lamberts S. W. (1994). Somatostatin-receptor scintigraphy in primary breast cancer.. Lancet.

[OCR_01052] van Roozendaal C. E., Klijn J. G., van Ooijen B., Claassen C., Eggermont A. M., Henzen-Logmans S. C., Foekens J. A. (1995). Transforming growth factor beta secretion from primary breast cancer fibroblasts.. Mol Cell Endocrinol.

